# Stability and Controlled Polymerization of Trithiocarbonate Chain Transfer Agents Under Harsh Conditions

**DOI:** 10.3390/polym17030297

**Published:** 2025-01-23

**Authors:** Thi Ngan Vu, Tomoya Nishimura, Yu Osaki, Toyohiro Otani, Shin-ichi Yusa

**Affiliations:** 1 Department of Applied Chemistry, Graduate School of Engineering, University of Hyogo, 2167 Shosha, Himeji 671-2280, Japan; vungan02091999@gmail.com (T.N.V.); nishitomo200101@gmail.com (T.N.); 2 Research & Development Center, R&D 2 Group, Ouchi Shinko Chemical Industrial Co., Ltd., 111 Shimojyukumae Sukagawa, Fukushima 962-0806, Japan; osaki@jp-noc.co.jp (Y.O.); otani@jp-noc.co.jp (T.O.)

**Keywords:** chain transfer agent, micelle, RAFT polymerization

## Abstract

This study investigates the stability and application of trithiocarbonate-based chain transfer agents (CTAs) in reversible addition–fragmentation chain transfer (RAFT) radical polymerization under harsh conditions. We evaluated the stability of 4-cyano-4-(2-carboxyethylthiothioxomethylthio) pentanoic acid (Rtt-17) and 4-cyano-4-(dodecylsulfanylthiocarbonyl) sulfanylpentanoic acid (Rtt-05) at 60 °C under basic conditions using ^1^H NMR and UV–vis absorption spectra, showing that Rtt-05 is more stable than Rtt-17. The greater stability of Rtt-05 is attributed to the hydrophobic dodecyl group, which allows it to form micelles in water, thereby protecting the trithiocarbonate group from the surrounding aqueous phase. In contrast, hydrophilic Rtt-17, without long alkyl chains, cannot form micelles in water. Following the stability assessment, Rtt-17 and Rtt-05 were employed for RAFT polymerization of hydrophilic monomers, such as *N,N*-dimethylacrylamide (DMA) and 2-(methacryloyloxy)ethyl phosphorylcholine (MPC). DMA can dissolve in both water and organic solvents, and MPC can dissolve in water and polar solvents. Both CTAs successfully controlled the polymerization of DMA, producing polymers with narrow molecular weight distributions (*M*_w_/*M*_n_) less than 1.2. Also, Rtt-17 demonstrated effective control of MPC polymerization, yielding *M*_w_/*M*_n_ values of around 1.2. However, during the polymerization of MPC, Rtt-05 failed to maintain control, resulting in a broad *M*_w_/*M*_n_ (≥1.9). The inability of Rtt-05 to control MPC polymerization is due to the formation of micelles, which disrupts the interaction between the hydrophilic MPC propagating radicals and the trithiocarbonate group in the hydrophobic core of Rtt-05 micelles. The findings provide critical insights into designing CTAs for specific applications, particularly for biomedical and industrial uses of hydrophilic polymers, highlighting the potential for precise molecular weight control and tailored polymer properties.

## 1. Introduction

Reversible addition–fragmentation chain transfer (RAFT) radical polymerization is a widely used method for preparing well-defined polymers with controlled molecular weights and narrow molecular weight distributions [1,2,3]. Chain transfer agents (CTAs) are essential to the RAFT process and play a crucial role in mediating the polymerization [4]. However, the effectiveness of RAFT polymerization is significantly influenced by the stability of these CTAs, especially when faced with the challenging conditions of polymerization reactions [5,6]. Thus, a deep understanding of how CTAs behave, particularly under adverse conditions, is vital for designing the RAFT technique and achieving superior polymer products.

**Figure 1:**
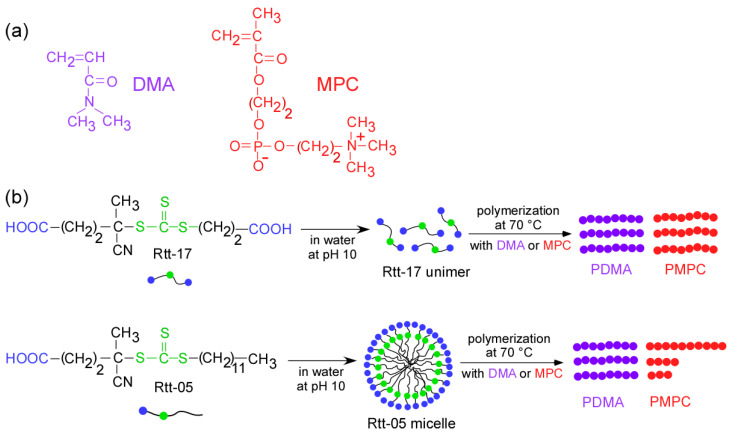
(**a**) Chemical structures of MPC and DMA and (**b**) conceptual illustrations of polymerization of the monomers under harsh conditions (pH 10 and 70 °C) using Rtt-17 and Rtt-05 as chain transfer agents.

Among the various types of CTAs, trithiocarbonate-based CTAs have received significant attention due to their efficiency in controlling polymer growth [7,8,9,10]. This study focused on the stability of two trithiocarbonate-type CTAs: 4-cyano-4-(2-carboxyethylthiothioxomethylthio)pentanoic acid (Rtt-17) and 4-cyano-4-(dodecylsulfanylthiocarbonyl)sulfanyl pentanoic acid (Rtt-05) (Figure 1). Vasilieva et al. [11] examined the stability of several CTAs with respect to temperature and pH and found them to be relatively stable under neutral and acidic conditions but sensitive to bases. Sogabe et al. [12] and Mertoglu et al. [13] also investigated the stability of CTAs, but only at a pH of around 6 and at 40 °C, which are relatively mild conditions. Previous studies examined the stability of CTAs under mild conditions, such as neutral and acidic environments. Still, comprehensive research on their stability under harsher conditions is lacking, particularly in basic and high-temperature environments. This study aimed to fill this gap by thoroughly and stematically investigating the stability of trithiocarbonate-based CTAs under basic conditions at 60 °C, which is a typical polymerization temperature in aqueous environments. There have been numerous studies investigating the stability of CTAs; however, to the best of our knowledge, for the first time, the stability of these trithiocarbonate-based CTAs was thoroughly and systematically investigated in our study under more harsh conditions, particularly basic conditions, and at 60 °C, which is a typical polymerization temperature in aqueous environments. The hydrolysis of the trithiocarbonate bond under strong basic conditions can significantly influence the properties of the resulting polymers [14,15,16].

In addition to studying the stability of these CTAs, this research also explored their application in the polymerization of hydrophilic monomers. Hydrophilic polymers are of great interest due to their wide range of applications, including in biomedical fields [17,18], water treatment [19,20], as functional materials [21,22], and as 3D-printed conducting polymers [23]. Applying RAFT polymerization to hydrophilic monomers using stable CTAs makes achieving precise control over the polymerization process possible, leading to polymers with desired properties for specific applications. In this context, we selected two hydrophilic monomers, 2-(methacryloyloxy)ethyl phosphorylcholine (MPC) and *N,N*-dimethylacrylamide (DMA), for controlled polymerization via RAFT. MPC is known for its biocompatibility and antifouling properties, making it an attractive monomer for biomedical applications [24,25]. On the other hand, due to its excellent solubility and reactivity, DMA is widely used in synthesizing hydrogels and other water-soluble polymers [26,27]. These monomers are chosen for their hydrophilic nature and potential applications in biomedical and industrial fields. In summary, this study aimed to investigate the stability of trithiocarbonate-based CTAs for the RAFT polymerization process using ^1^H NMR and UV–vis spectroscopy and their application for controlled radical polymerization of hydrophilic monomers such as MPC and DMA. The findings of this study will contribute to the advancement of RAFT polymerization and the development of innovative polymeric materials.

## 2. Materials and Methods

### 2.1. Materials

4-Cyano-4-(2-carboxyethylthiothioxomethylthio)pentanoic acid (Rtt-17) and 4-cyano-4-(dodecylsulfanylthiocarbonyl)sulfanyl pentanoic acid (Rtt-05), gifted from Ouchi Shinko Chemical Industrial (Tokyo, Japan), and 2-(methacryloyloxy)ethyl phosphorylcholine (MPC), gifted from NOF (Tokyo, Japan), were used without further purification. Nile red (≥97%) from Sigma-Aldrich (St. Louis, MO, USA) and 4,4′-azobis(4-cyanovaleric acid) (V-501, 95%) from Fujifilm Wako Pure Chemical (Osaka, Japan) were used without further purification. *N*,*N*-Dimethylacrylamide (DMA, ≥98%) purchased from Fujifilm Wako Pure Chemical (Osaka, Japan) was distilled under reduced pressure prior to use to remove polymerization inhibitors.

### 2.2. Measurements

^1^H NMR measurements were performed using a JEOL (Tokyo, Japan) JNM-ECZ 400 MHz spectrometer. UV–vis absorption spectra were recorded on a Jasco (Tokyo, Japan) V-730 UV–vis spectrophotometer. Gel-permeation chromatography (GPC) measurements were used to determine the number-average molecular weights (*M*_n_) and molecular weight distributions (*M*_w_/*M*_n_). GPC curves were obtained using a Tosoh RI-8020 refractive index (RI) detector working at 40 °C with a Shodex (Tokyo, Japan) GF-7M column, a Tosoh (Tokyo, Japan) DP-8020 pump, and a mixture of 50 mM phosphate buffer at pH 9 and acetonitrile (9/1, *v*/*v*) as an eluent. Using a calibration made from standard sodium poly(styrenesulfonate), the *M*_n_ and *M*_w_/*M*_n_ were estimated. Dynamic light scattering (DLS) measurements were performed using a Malvern (Worcestershire, UK) Zetasizer Nano ZS with a He–Ne laser.

### 2.3. Stability of CTAs and Determination of Critical Micelle Concentration (CMC)

CTAs were dissolved in D_2_O at a concentration of 10 g/L, and then the pH levels of the solutions were adjusted using NaOD and DCl. The aqueous solutions were heated at 60 °C for 24 h, then cooled to room temperature. To confirm the decomposition of the CTAs, ^1^H NMR spectra were obtained with concentrations of approximately 10 g/L (32.5 mM for Rtt-17 and 24.8 mM for Rtt-05) for each CTA, and UV–vis absorption spectra were obtained at a concentration of 0.025 g/L (0.08 mM for Rtt-17 and 0.0625 mM for Rtt-05). UV–vis absorption spectra were used to evaluate the critical micelle concentrations (CMCs) for Rtt-17 and Rtt-05 at pH 11 and 25 °C using Nile red as a hydrophobic dye. Rtt-17 and Rtt-05 aqueous solutions were prepared at varying concentrations ranging from 0.1 g/L to 10 g/L. Nile red with 1 μM was added to the aqueous Rtt-17 and Rtt-05 solutions.

### 2.4. RAFT Polymerization of N,N-Dimethylacrylamide (DMA)

To study polymerization kinetics, DMA was polymerized via RAFT using Rtt-17 and Rtt-05 as CTAs. The target degree of polymerization (DP) was 50. A typical example was as follows. DMA (0.49 g, 5 mmol), a CTA (0.1 mmol), and V-501 (11.2 mg, 0.04 mmol) with a feeding molar ratio of [DMA]/[CTA]/[V-501] = 50/1/0.4 were dissolved in 5 mL D_2_O at pH 10 and then transferred to an NMR tube. The polymerization solution was degassed by purging with Ar gas for 5 min before polymerization at 70 °C. The percentage conversion (*p*) of monomers was confirmed by ^1^H NMR before and after the corresponding reaction time (Figure S4). The *p* value was estimated from integral intensity ratios of the vinyl protons of DMA at 6.51 ppm and the pendant methyl protons at 2.98 ppm.

### 2.5. RAFT Polymerization of 2-(Methacryloyloxy)ethyl Phosphorylcholine (MPC)

To study polymerization kinetics, MPC was polymerized via RAFT using Rtt-17 and Rtt-05 as CTAs. The target DP was 50. A typical example was as follows. MPC (1.48 g, 5 mmol), a CTA (0.1 mmol), and V-501 (11.2 mg, 0.04 mmol) with a feeding molar ratio of [MPC]/[CTA]/[V-501] = 50/1/0.4 were dissolved in 5 mL D_2_O at pH 10 and then transferred to an NMR tube. The polymerization solution was degassed by purging with Ar gas for 5 min before polymerization at 70 °C. The *p* value was confirmed by ^1^H NMR before and after the corresponding reaction time (Figure S3). The *p* value was estimated from integral intensity ratios of the vinyl protons of MPC at 5.98 ppm and the pendant methylene proton at 3.04 ppm.

## 3. Results and Discussion

### 3.1. Stability of CTAs Under Basic Conditions at 60 °C

Due to the carboxylic acid group in the chemical structures of Rtt-17 and Rtt-05 as CTAs, they can dissolve in basic aqueous solutions. Generally, the trithiocarbonate structure is not stable against hydrolysis in water [28,29]. The chemical stability was measured using ^1^H NMR for Rtt-17 and Rtt-05 at 60 °C in basic aqueous solutions, which are typical conditions of RAFT polymerization of hydrophilic monomers in water. Rtt-17 and Rtt-05 were dissolved in D_2_O at a concentration of 10 g/L, and the pH was adjusted using NaOD. The solution was then heated at 60 °C for 24 h, cooled to room temperature, and measured by ^1^H NMR (Figures S1 and S2). For Rtt-17, the methyl proton signal near trithiocarbonate was observed at 1.74 ppm. In the case of Rtt-05, the signal at 0.74 ppm attributed to the methyl protons in the dodecyl group was observed, and the methyl proton signal near trithiocarbonate was observed at 1.74 ppm. The degradation behavior of Rtt-17 and Rtt-05 was evaluated to monitor the ^1^H NMR signal intensity at 1.74 ppm at 60 °C for 24 h with varying pH values ranging from 9 to 13. A decrease in the methyl proton peak at 1.74 ppm of Rtt-17 was observed at pH > 11. Thus, the trithiocarbonate group in Rtt-17 underwent hydrolysis at pH > 11. In contrast, a decrease in the methyl proton peak at 1.74 ppm of Rtt-05 was observed at pH > 12, indicating that the trithiocarbonate was hydrolyzed under this pH condition. The trithiocarbonate group of Rtt-17 degraded at a lower pH than that of Rtt-05. Due to the hydrophilic carboxylate ion and the hydrophobic dodecyl group, Rtt-05 may form micelles in water. Because the trithiocarbonate group may exist in the hydrophobic core of the micelle, it is less affected by the base in the aqueous phase. In contrast, since Rtt-17 does not have significant hydrophobic groups, it can dissolve in water with a single molecular state without association. Therefore, Rtt-17 is less stable against bases than Rtt-05 because Rtt-17 is affected directly by hydroxide ions in water.

**Figure 2:**
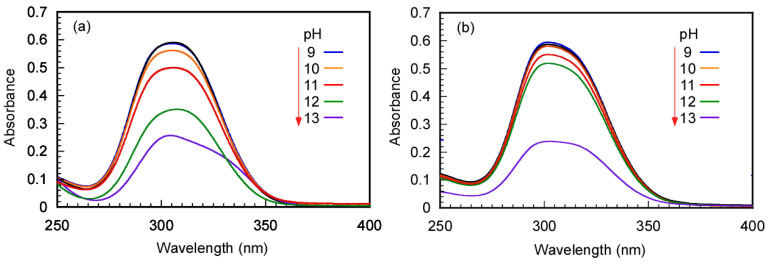
UV–vis absorption spectra of (**a**) Rtt-17 and (**b**) Rtt-05 with 0.025 g/L at 25 °C under various pH conditions after 24 h at 60 °C: The pH values were indicated in the spectra.

The stability and decomposition of Rtt-17 and Rtt-05 under basic and 60 °C conditions were also studied using UV–vis absorption spectra (Figure 2). The UV–vis absorption peak at 309 nm attributed to the trithiocarbonate group was monitored. The CTA aqueous solutions were heated at 60 °C for 24 h at varying pH values. After that, UV–vis absorption spectra were measured at 25 °C. The absorbance at 309 nm for Rtt-17 decreased with increasing pH values; degradation began to occur at pH > 11, suggesting decomposition of the trithiocarbonate group at pH > 11. In contrast, for Rtt-05, a sharp degradation of the peak at 309 nm was observed at pH > 12, indicating that Rtt-05 decomposes at pH > 12.

**Figure 3:**
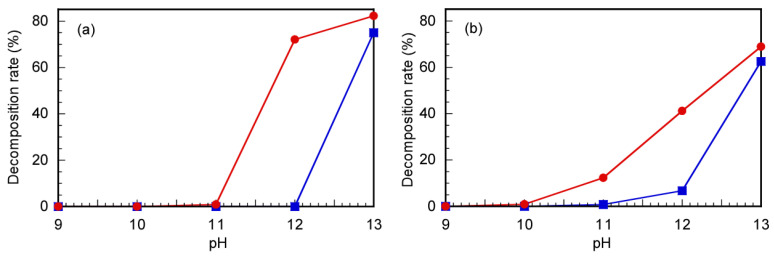
The decomposition rates estimated from (**a**) ^1^H NMR integral intensity ratios of signals at 1.74 ppm and (**b**) UV–vis absorption spectra for Rtt-17 (●) and Rtt-05 (■) under various pH conditions after 24 h at 60 °C.

The CTA decomposition rates were summarized under varying pH conditions at 60 °C for 24 h, estimated via ^1^H NMR and UV–vis absorption spectroscopy (Figure 3). The signal intensity of the methyl proton at 1.74 ppm, which is located near the trithiocarbonate group, decreased at different pHs and 60 °C, as shown in Figure 3a. For Rtt-17, the methyl proton signal at 1.74 ppm decreased by 74.5% from the initial intensity at pH > 11, whereas for Rtt-05, this signal showed no change at pH ≤ 12 and only a 71.2% decrease was observed at pH > 12. In addition, the absorption degradation at 309 nm, corresponding to the trithiocarbonate moiety, is presented in Figure 3b. The absorption spectrum decomposition rate of Rtt-17 started to exhibit a 16.7% decrease at pH 11, and the decline accelerated sharply at pH > 11, whereas Rtt-05 could exhibit stable absorption at pH ≤ 11, indicating that it remained stable under these conditions. The results demonstrated that Rtt-05 showed higher stability under basic conditions than Rtt-17, mainly due to its ability to form micelles that provide a protective effect to the trithiocarbonate group. The formation of micelles by amphiphilic molecules like Rtt-05 has been well-documented to enhance the stability of sensitive groups by creating a microenvironment that isolates them from reactive species in bulk solutions [30].

### 3.2. Micelle Formation

From the stability test, Rtt-05 was more stable than Rtt-17 at 60 °C under basic conditions. Rtt-05 shows an amphiphilic nature due to the hydrophilic carboxylate anion and the hydrophobic dodecyl group. In basic water, Rtt-05 may form micelles, so the trithiocarbonate group was protected from the outer water phase. In contrast, hydrophilic Rtt-17 dissolved in water as a single molecular state because Rtt-17 does not have hydrophobic alkyl chains. UV–vis absorption spectra were used to evaluate the critical micelle concentration (CMC) for Rtt-17 and Rtt-05 at pH 10 using Nile red as a hydrophobic dye. The absorption spectrum of Nile red was affected by the surrounding environment. Nile Red absorbs in the visible wavelength region. In water, the dye is almost insoluble. In the presence of surfactants, Nile red dissolves, resulting in a change in absorption intensity and colors [31,32] because the hydrophobic Nile red is encapsulated in the hydrophobic core of the surfactant micelles and dissolves in water.

**Figure 4:**
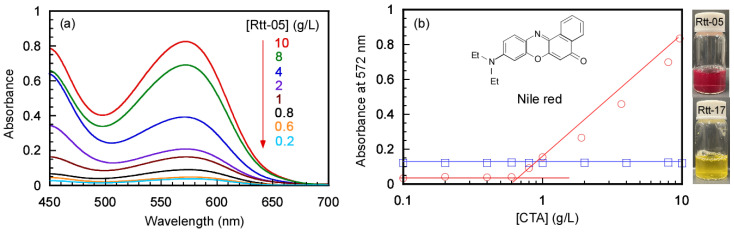
(**a**) UV–vis absorption spectra of Nile red in water in the presence of Rtt-05 at various concentrations at pH 11: The concentration of Rtt-05 ([Rtt-05]) was indicated in the spectra and (**b**) absorbance at 572 nm for Nile red as a function of CTA concentrations at pH 11 for Rtt-17 (**□**) and Rtt-05 (**○**) at 25 °C.

The maximum absorbance of Nile red can be observed at 572 nm in the presence of 10 g/L of Rtt-05 in water at pH 10. To determine the CMC values, UV–vis absorption spectra of Nile red in the presence of Rtt-17 and Rtt-05 were measured at varying CTA concentrations. The plots between the absorbance at 572 nm and the CTA concentrations were evaluated (Figure 4a). With increasing Rtt-05 concentrations, the red color of Nile red can be observed to increase the absorbance at 572 nm (Figure 4b). In the plots of the absorbance and the CTA concentrations, the intersection of the two tangent lines corresponds to the CMC value. The CMC value of Rtt-05 was determined to be 0.7 g/L (1.73 mM). For Rtt-17, the solution could not dissolve the Nile red in water, even at high CTA concentrations, so the absorption of Nile red could not be observed (Figure S5). This observation indicates that Rtt-17 cannot form micelle structures in water because Rtt-17 has two hydrophilic carboxylate groups without hydrophobic parts. The CMC values for sodium laurate (C12) and sodium stearate (C18) are 25.5 mM at 25 °C [33] and 0.8 mM at 60 °C [34], respectively. The solubility of sodium stearate in water at 25 °C is too low. Therefore, the CMC for sodium stearate is measured at 60 °C. The CMC (0.7 g/L, 1.73 mM) of Rtt-05 was between the CMCs of sodium laurate and sodium stearate.

**Figure 5:**
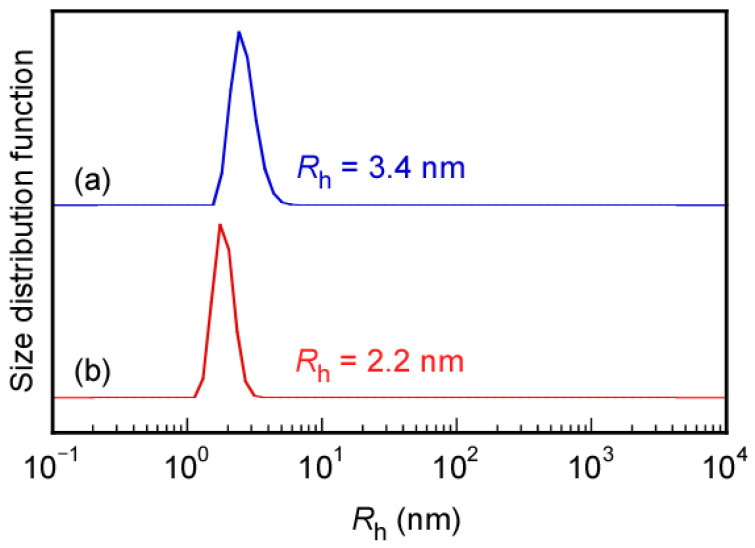
Hydrodynamic radius (*R*_h_) distributions for Rtt-05 at 10 g/L in water under pH 10 at (**a**) 25 °C and (**b**) 70 °C.

The formation of micelle structures by Rtt-05 above the CMC was confirmed through Nile red uptake experiments in water at 25 °C and pH 11. Generally, to start radical polymerization with an azo initiator, the reaction temperature is raised to 60–70 °C, which breaks the C-N bond in the azo initiator and generates primary radicals. We studied the formation of micelles by Rtt-05 at 70 °C, a temperature at which radicals were generated during the initiation process of radical polymerization. This experiment was conducted without adding an initiator because the purpose was to evaluate the aggregation behavior of Rtt-05. Nile red can be dissolved in water at 70 °C at the concentration of Rtt-05 ([Rtt-05]) = 10 g/L (24.8 mM). Therefore, at 70 °C, in water, at pH 10, Rtt-05 formed micelles, incorporating and solubilizing the hydrophobic Nile red in the core of the micelles. Furthermore, to confirm micelle formation of Rtt-05 at 10 g/L (24.8 mM), which is higher than the CMC (0.7 g/L, 1.73 mM), dynamic light scattering (DLS) measurements were performed in water at pH 10 at both 25 °C and 70 °C (Figure 5). The hydrodynamic radii (*R*_h_) of Rtt-05 at 25 °C and 70 °C were 3.4 nm and 2.2 nm, respectively. These large *R*_h_ values suggested that Rtt-05 micelles were formed at both 25 °C and 70 °C.

### 3.3. Polymerization Kinetics for CTAs

Rtt-17 has two carboxylate groups and no hydrophobic parts, so it does not aggregate in water at pH 10. On the other hand, Rtt-05 has a hydrophilic carboxylate group and a hydrophobic dodecyl group, so it forms micelles in water at pH 10. We studied the differences in the polymerization mechanisms of these two trithiocarbonate-type CTAs, Rtt-17 and Rtt-05. The hydrophilic V-501, which has two carboxylate groups, was used as an initiator. The DMA monomer, which is soluble in both water and organic solvents, and the MPC monomer, which is soluble only in water, were used as the monomers for the polymerization kinetic study.

**Figure 6:**
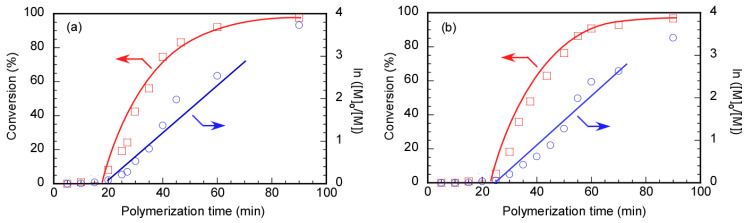
Time–conversion (*p*) (**□**) and first-order kinetic plots (**○**) of PDMA using (**a**) Rtt-17 and (**b**) Rtt-05; [M]_0_ and [M] are the monomer concentrations at the polymerization time = 0 min and the corresponding time, respectively: The arrows indicated the axis.

**Figure 7:**
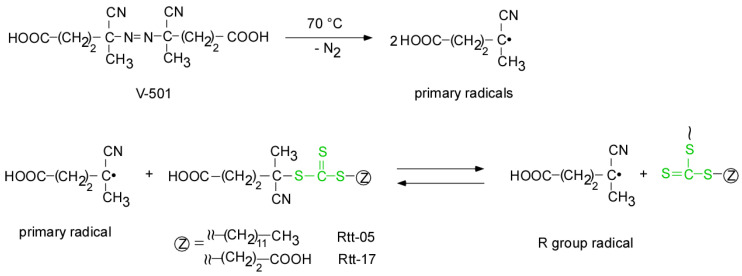
The early-stage, pre-equilibrium reaction between V-501 and CTAs.

The RAFT polymerization of DMA was carried out using Rtt-17 and Rtt-05, with a target DP of 50. The polymerization was performed under an Ar atmosphere at 70 °C in D_2_O at pH 10, with a feeding molar ratio of [DMA]/[CTA]/[V-501] = 50/1/0.4. The percentage conversion (*p*) at different polymerization times was estimated by ^1^H NMR. Under these polymerization conditions, Rtt-17 and Rtt-05 underwent almost no degradation. While Rtt-17 dissolves molecularly in water, Rtt-05 forms micelles. The trithiocarbonate group of Rtt-05 is located within the hydrophobic micelle core. Although DMA is soluble in water, it can also dissolve in organic solvents, allowing it to penetrate the micelle core formed by Rtt-05. The induction periods of Rtt-17 and Rtt-05 were observed from the relationship between the polymerization time and *p* (Figure 6). The induction periods of Rtt-17 and Rtt-05 were 19 min and 22 min, respectively. At the early stage of polymerization, heating causes V-501 to cleave and generate primary radicals. The structure of the primary radical generated from V-501 is identical to that of the “R” radical generated from Rtt-17 and Rtt-05 (Figure 7). The primary radicals generated from V-501 achieve a pre-equilibrium state, undergoing reversible addition and fragmentation reactions with the trithiocarbonate of the CTA. This reaction period is assumed to have been observed as the induction period. Subsequently, polymerization starts with the reaction between the R radical from the CTA and DMA, leading to an increase in the conversion. After 90 min of polymerization, the *p* values of Rtt-17 and Rtt-05 were 97.2% and 91.2%, respectively. The first-order plot, which increased linearly in the early polymerization stages, suggested a constant concentration of propagating radicals. The relationship between the polymerization time and the *p* value of DMA, as well as the first-order plot, were almost the same for Rtt-17 and Rtt-05. Rieger et al. [4] investigated the controlled polymerization of DMA using a trithiocarbonate CTA in an organic solvent at a temperature of 70 °C. The results indicated that the process produced well-controlled polymers with respect to molecular weight, molecular weight distribution, and chain length. In this study, we highlight the novelty of selecting appropriate CTAs for the polymerization of hydrophilic monomers in an aqueous environment under challenging conditions.

**Figure 8:**
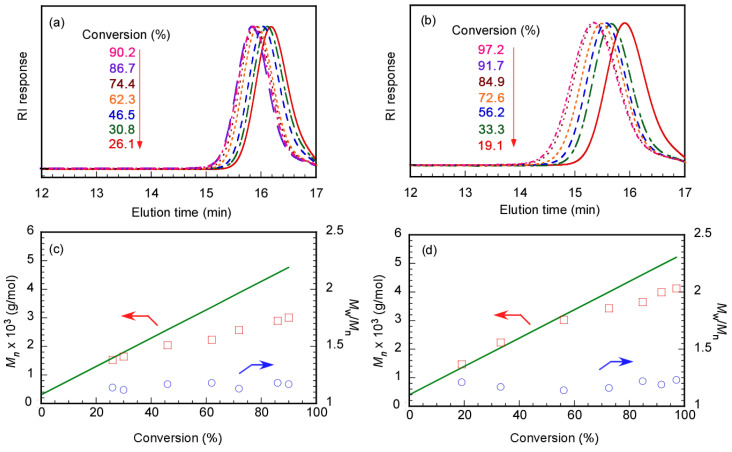
GPC elution curves of PDMA prepared using (**a**) Rtt-17 and (**b**) Rtt-05 and conversion (*p*)–*M*_n_ (**□**) with theoretical line (**—**) and *p*-*M*_w_/*M*_n_ plots (**○**) using (**c**) Rtt-17 and (**d**) Rtt-05: The arrows indicated the axis.

We performed GPC measurements to confirm the polymerization kinetics of DMA in water at pH 10 using Rtt-17 and Rtt-05. The target DP was 50. Rtt-05 formed micelles in the polymerization system because the concentration of Rtt-05 was 8.07 g/L (20 mM), which is higher than the CMC (0.7 g/L, 1.73 mM). On the other hand, Rtt-17 was dissolved in water without aggregation. The *M*_n_ and *M*_w_/*M*_n_ values estimated from GPC were plotted against *p* using Rtt-17 and Rtt-05 (Figure 8). In both Rtt-17 and Rtt-05, the *M*_n_ values increased with *p* and were relatively close to the theoretical *M*_n_ (*M*_n_(theo)) values. The *M*_n_(theo) values were calculated using the following equation:

(1) $$M_{n}\left(theo\right)=\frac{{\left[M\right]}_{0}}{{\left[CTA\right]}_{0}}\times \frac{p}{100}\times M_{M}+M_{CTA}$$

where [M]_0_ and [CTA]_0_ are the initial concentrations of the monomer and the CTA and *M*_M_ and *M*_CTA_ are the molecular weights of the monomer and the CTA, respectively. The *M*_w_/*M*_n_ values indicated a narrow range of 1.14−1.21 independent of *p*. These observations suggest that the polymerization of DMA can be controlled by either Rtt-17 or Rtt-05. The DMA monomer can dissolve water and various organic solvents. Therefore, if Rtt-05 forms micelles in water, DMA can penetrate the core to contact with the trithiocarbonate group in Rtt-05.

**Figure 9:**
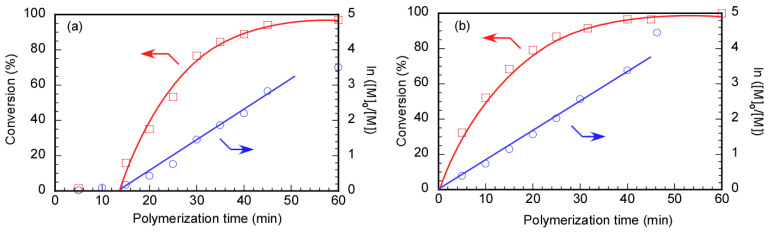
Time–conversion (*p*) (**□**) and first-order kinetic plots (**○**) of PMPC using (**a**) Rtt-17 and (**b**) Rtt-05; [M]_0_ and [M] are the monomer concentrations at the polymerization time = 0 min and the corresponding time, respectively: The arrows indicated the axis.

The relationship between the polymerization time and *p*, along with the first-order kinetic plot, was studied during the RAFT polymerization of MPC in D_2_O at pH 10 using Rtt-17 and Rtt-05 (Figure 9). Due to its high hydrophilicity, MPC could not penetrate the hydrophobic core of the Rtt-05 micelles. RAFT polymerization was performed under an Ar atmosphere at 70 °C in water at pH 10, with a feeding molar ratio of [MPC]/[CTA]/[V-501] = 50/1/0.4. An induction period of 14 min with 0% conversion was observed for polymerization using Rtt-17. The induction period can often be observed for RAFT polymerization. The induction period is caused by the pre-equilibrium state formed through reversible addition and fragmentation reactions between the trithiocarbonate of Rtt-17 and the primary radicals generated from the initiator, V-501. The polymerization of MPC is initiated by the R radical generated from Rtt-17. The MPC conversion reached 95.2% after 1 h of polymerization. Additionally, in the early stages of the first-order kinetic plot using Rtt-17, the linear line suggests that the concentration of propagating radicals during polymerization was constant. On the other hand, no induction period was observed in the relationship between the polymerization time and *p* of MPC using Rtt-05. The straight line passing through the origin for the first-order kinetic plot using Rtt-05 indicates that the concentration of propagating radicals was constant during the polymerization. The RAFT polymerization of MPC showed the induction period using Rtt-17, while no induction period was observed using Rtt-05. This finding suggests that the polymerization mechanisms of the MPC of Rtt-17 and Rtt-05 were different.

**Figure 10:**
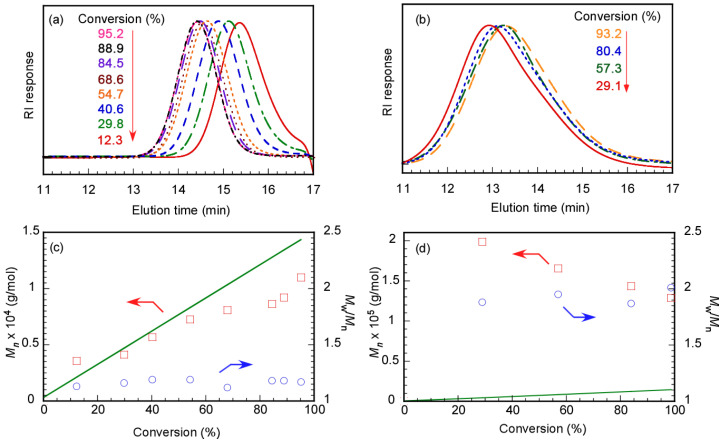
GPC elution curves of PMPC prepared using (**a**) Rtt-17 and (**b**) Rtt-05 and conversion (*p*)–*M*_n_ (**□**) with theoretical line (**—**) and *p*-*M*_w_/*M*_n_ plots (**○**) using (**c**) Rtt-17 and (**d**) Rtt-05: The arrows indicated the axis.

Polymerization of MPC in water at pH 10 was performed using Rtt-17, and GPC was measured at various *p* values to study the polymerization mechanism with the target DP = 50. MPC, Rtt-17, and V-501 were fully dissolved in water at pH 10. As the *p* value of the MPC increased, the GPC elution time became shorter (Figure 10). Plots of *p* versus *M*_n_ (and *M*_w_/*M*_n_) estimated from GPC were prepared. As *p* increased, *M*_n_ increased close to the *M*_n_(theo) value. Additionally, *M*_w_/*M*_n_ remained below 1.20, showing a narrow range independent of *p*. These observations indicate that polymerization of MPC proceeded via a controlled mechanism. During the initial stage of polymerization in water, reversible addition and fragmentation reactions between Rtt-17 and V-501 took place, after which MPC propagating radicals reacted with the trithiocarbonate, allowing for controlled polymerization. Essentially, this demonstrates that conventional RAFT polymerization occurred.

MPC was polymerized in water at pH 10 with Rtt-05, and GPC was measured at different conversions with the target DP = 50. In the RAFT process, to control the polymerization, the trithiocarbonate in Rtt-05 must encounter the MPC propagating radicals for reversible addition and fragmentation reactions. The *M*_n_ values of PMPC were larger than the *M*_n_(theo) values, independent of *p* (Figure 10). When *p* was 93.2%, the *M*_n_ was 1.28 × 10^5^ g/mol, while the *M*_n_(theo) was 1.45 × 10^4^ g/mol, showing a difference of 8.8 times. The relationship between *M*_n_ and *p* exhibited the behavior of conventional radical polymerization rather than controlled radical polymerization. Furthermore, *M*_w_/*M*_n_ showed large values of 1.89–2.01, independent of *p*. These results suggest that the polymerization of MPC in water could not be effectively controlled with Rtt-05. Under the polymerization conditions, Rtt-05 formed micelles. It is difficult for MPC to penetrate the hydrophobic core of Rtt-05 micelles due to its large size and high polarity. As a result, the trithiocarbonate of Rtt-05 cannot react with MPC. Since V-501 is water-soluble, it can initiate the polymerization of MPC in the aqueous phase, resulting in the progression of conventional radical polymerization without regard to Rtt-05. We have confirmed that MPC can be polymerized in water via conventional free radical polymerization without Rtt-05.

Although Rtt-05 can effectively control the DMA polymerization, it failed to control the MPC polymerization (Table 1). This can be attributed to several factors. Firstly, the differences in the hydrophilicity/hydrophobicity balance of the monomers play a crucial role. DMA is a less hydrophilic monomer compared to MPC, making it more compatible with the hydrophobic core of Rtt-05 micelles. In the case of DMA, Rtt-05 can effectively participate in the polymerization process because DMA can penetrate into the Rtt-05 micelles, allowing for better control over the reaction. On the other hand, the polymerization of MPC cannot be controlled via Rtt-05 because the pendant hydrophilic charge groups in the MPC prevent them entering the inside of the hydrophobic core of Rtt-05 micelles. Therefore, the trithiocarbonate group in Rtt-05, which is a key structure of RAFT polymerization, cannot interact with MPC. Rtt-05 formed micelles in aqueous solutions due to its hydrophilic carboxylate anion and hydrophobic dodecyl group. While micelle formation can protect the trithiocarbonate group from degradation, it may hinder the RAFT polymerization process by preventing effective interaction between the CTA and the propagating radicals, leading to a broader *M*_w_/*M*_n_ and a deviation in *M*_n_ of the obtained PMPC. Bhuchar et al. [35] demonstrated the ability to control the polymerization of MPC in methanol at 70 °C using dithiocarbonate CTAs and trithiocarbonate CTAs. They found that the molecular weight could be effectively controlled when employing either the dithio-based or trithio-based CTAs. However, the polydispersity was better managed when using the dithio-based chain transfer agent.

These outcomes offer valuable guidance for designing and selecting stable CTAs in RAFT polymerization, especially when applied to hydrophilic monomers in aqueous environments to achieve controlled polymerization and high-quality polymer products.

## 4. Conclusions

We studied the stability and RAFT polymerization effectiveness of two trithiocarbonate-based CTAs, Rtt-17 and Rtt-05, under harsh conditions. The results demonstrated that Rtt-05 exhibited superior stability at 60 °C and pH ≥ 10 due to its hydrophobic dodecyl group, facilitating micelle formation. However, this micelle formation hindered its ability to control the polymerization of highly hydrophilic monomers like MPC, leading to a broad *M*_w_/*M*_n_. In contrast, Rtt-17 effectively controlled the polymerization of both MPC and DMA, producing polymers with a narrow *M*_w_/*M*_n_. These insights offer valuable guidance for designing and selecting CTAs in RAFT polymerization, especially when applied to hydrophilic monomers in aqueous environments to achieve controlled polymerization and high-quality polymer products.

## Figures and Tables

**Table 1:** Stability and polymerization controlled under different conditions.

Study	Conditions	Stability of Trithiocarbonate-Based CTAs	Polymerization Control of DMA	Polymerization Control of MPC
Vasilieva et al. [11]	Neutral, acidic	Stable	Not studied	Not studied
Sogabe et al. [12]	pH~6, 40 °C	Stable	Not studied	Not studied
Mertoglu et al. [13]	pH~6, 50 °C	Stable	Not studied	Not studied
This study	Basic, 60 °C	Less stable, hydrolyzes at pH > 11	Effective control, narrow *M*_w_/*M*_n_	Rtt-05 with micelle structure ineffective control, broad *M*_w_/*M*_n_, Rtt-07 with single state well-controlled, narrow *M*_w_/*M*_n_

## Data Availability

The original contributions presented in the study are included in the article/Supplementary Material; further inquiries can be directed to the corresponding author.

## Supplementary Materials

The following supporting information can be downloaded at https://www.mdpi.com/article/10.3390/polym17030297/s1: Figure S1. ^1^H NMR spectra for Rtt-17 with 10 g/L in D_2_O before (a) and after heating at 60 °C for 24 h at pH 9 (b), 10 (c), 11 (d), 12 (e), and 13 (f). Figure S2. ^1^H NMR spectra for Rtt-05 with 10 g/L in D_2_O before (a) and after heating at 60 °C for 24 h at pH 9 (b), 10 (c), 11 (d), 12 (e), and 13 (f). Figure S3. ^1^H NMR spectra for MPC polymerization system (a) before and (b) after heating for 60 min via RAFT in D_2_O at 25 °C. Figure S4. ^1^H NMR spectra for DMA polymerization system (a) before and (b) after heating for 90 min via RAFT in D_2_O at 25 °C. Figure S5. UV–vis absorption spectrum of Nile red in water in the presence of Rtt-17 at 10 g/L at pH 11.
