# Supplementary material for: Stability and Controlled Polymerization of Trithiocarbonate Chain Transfer Agents Under Harsh Conditions
Source: Polymers (Basel). 2025 Jan 23;17(3):297. doi: 10.3390/polym17030297 (PMC11820548; doi:10.3390/polym17030297)
Supplement: Supplementary file 1 [file polymers-17-00297-s001.zip › polymers-3431254-supplementary.pdf]

Supplementary information

Stability and Controlled Polymerization of Trithiocarbonate Chain Transfer Agents Under Harsh Conditions

Thi Ngan Vu[1], Tomoya Nishimura[1], Yu Osaki[2], Toyohiro Otani[2], and Shin-ichi Yusa[1,*]

[1]Department of Applied Chemistry, Graduate School of Engineering, University of Hyogo, 2167 Shosha, Himeji 671-2280, Hyogo, Japan; vungan02091999@gmail.com (T.N.V.); nishitomo200101@gmail.com (T.N.)

[2]Research & Development Center R&D 2 Group, Ouchi Shinko Chemical Industrial Co. LTD., 111 Shimojyukumae Sukagawa, Fukushima 962-0806, Japan; osaki@jp-noc.co.jp (Y.O.); otani@jp-noc.co.jp (T.O.)

Correspodence: yusa@eng.u-hyogo.ac.jp

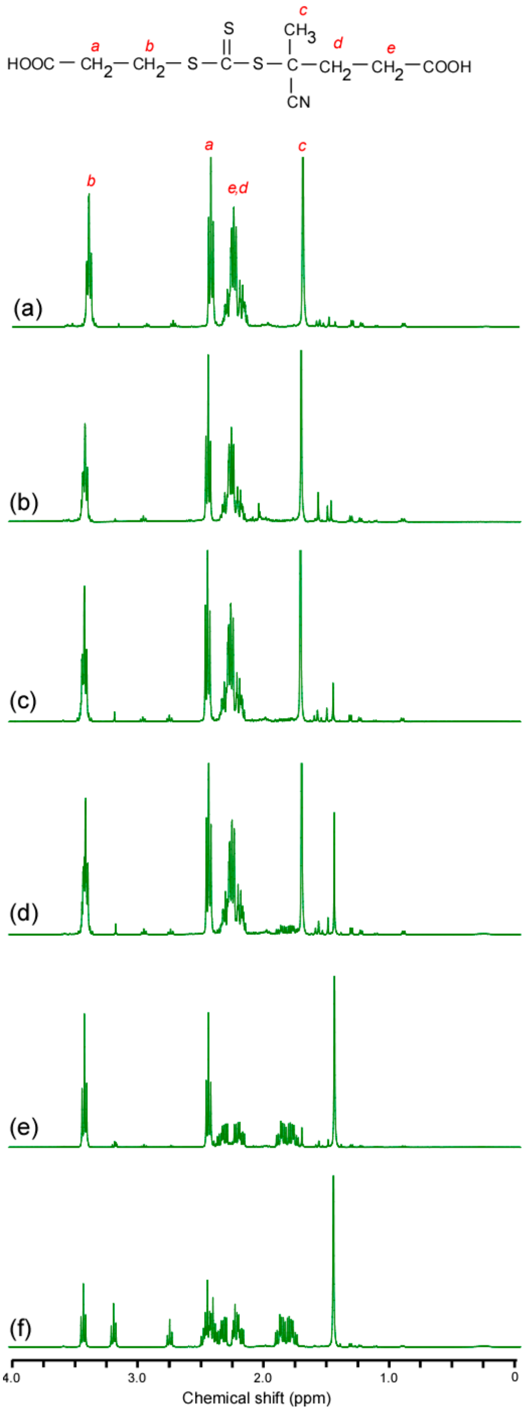

Figure S1. ^{1}H NMR spectra for Rtt-17 with 10 g/L in D_2O before (a) and after heating at 60 °C for 24 h at pH 9 (b), 10 (c), 11 (d), 12 (e), and 13 (f).

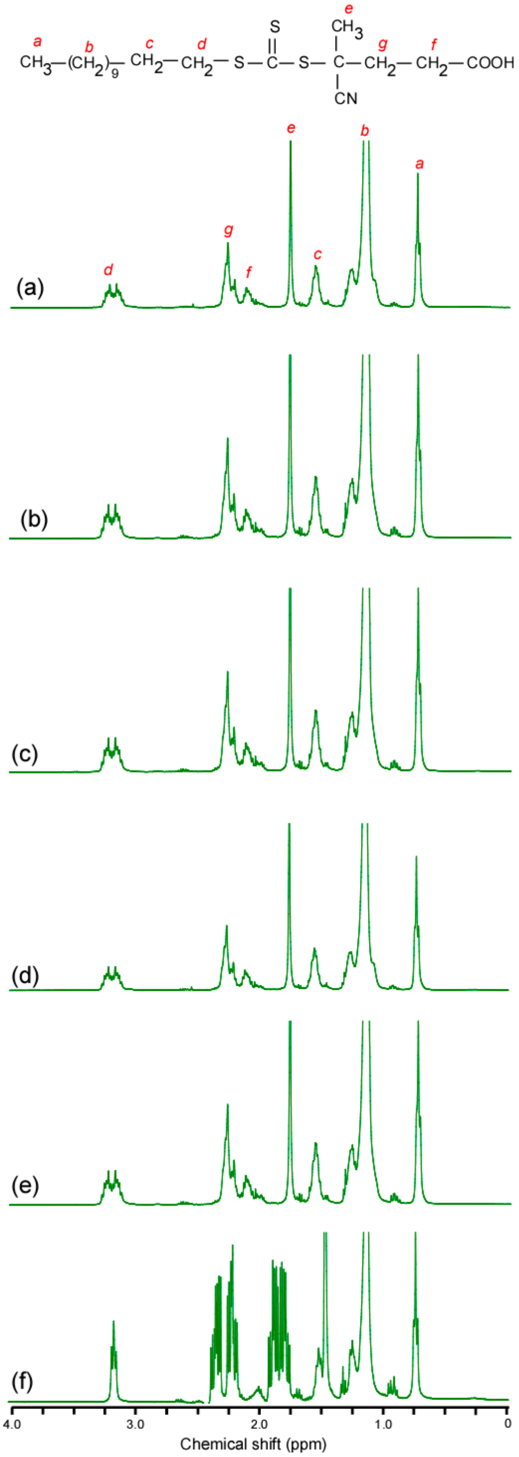

Figure S2. ^{1}H NMR spectra for Rtt-05 with 10 g/L in D_2O before (a) and after heating at 60 °C for 24 h at pH 9 (b), 10 (c), 11 (d), 12 (e), and 13 (f).

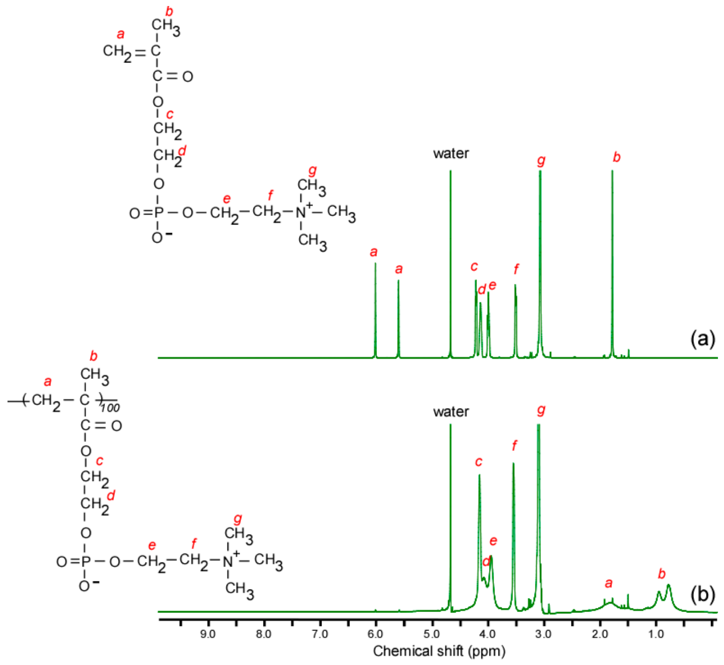

Figure S3. ^{1}H NMR spectra for MPC polymerization system (a) before and (b) after heating for 60 min via RAFT in D_2O at 25 °C.

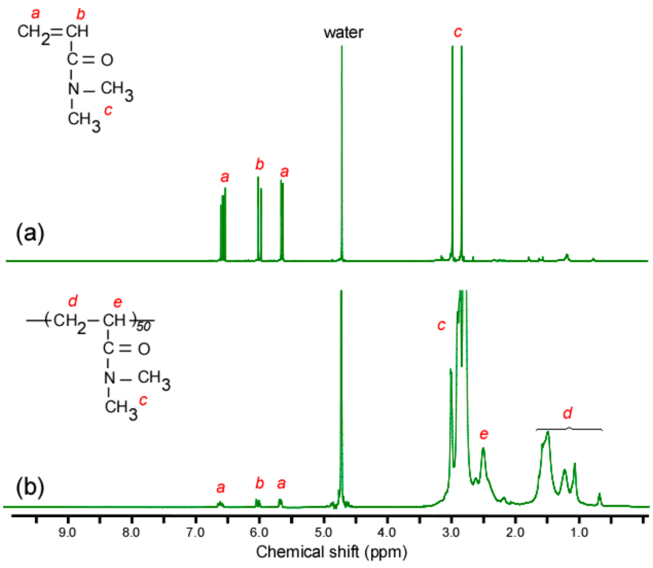

Figure S4. ^{1}H NMR spectra for DMA polymerization system (a) before and (b) after heating for 90 min via RAFT in D_2O at 25 °C.

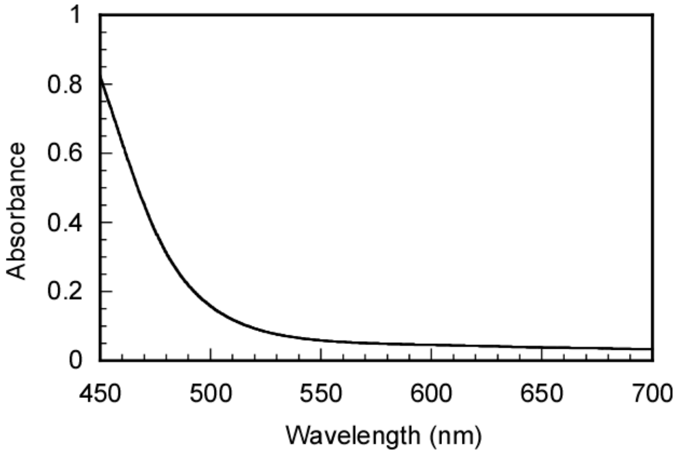

Figure S5. UV–vis absorption spectrum of Nile red in water in the presence of Rtt-17 at 10 g/L at pH 11.
